# Rotate-on-Retract Procedural Automation for Robotic-Assisted Percutaneous Coronary Intervention: First Clinical Experience

**DOI:** 10.1155/2018/6086034

**Published:** 2018-12-20

**Authors:** Arif Al Nooryani, Wael Aboushokka

**Affiliations:** Al Qassimi Hospital, Sharjah, Dubai, UAE

## Abstract

The advent of percutaneous coronary intervention (PCI) has dramatically changed the outlook for patients with cardiovascular disease. However, room for improvement and advancement remains in the safety, speed, and efficiency of manually guided PCI. In recent years, the CorPath robotic platform (Corindus Inc., Waltham, MA) has been approved to aid the interventionalist during PCI and other endovascular interventions. Favorable results in several clinical studies suggest that robotic-assisted PCI may further improve patient outcomes while also benefiting the interventionalist through reduced orthopedic strain and less exposure to ionizing radiation. In this report, we communicate our experience with the first-in-human use of a new, optional automation feature that has been added to the platform's guidance software. This “Rotate-on-Retract” feature is designed to facilitate faster and more precise maneuvering of the guidewire through tortuous vessels by automatically rotating the guidewire whenever it is retracted by the operator. This movement changes the tip's orientation in preparation for the next advancement. We evaluated this feature in a patient undergoing PCI to treat a severe (90% stenotic), long, diffuse, and calcified lesion of the proximal to mid LAD segments.

## 1. Introduction

The advent of percutaneous approaches to the treatment of obstructive coronary disease more than 40 years ago triggered a foundational shift in the management and outcomes of cardiovascular disease [1]. Since the first successful balloon dilatation angioplasty, the field of percutaneous coronary intervention (PCI) has witnessed the evolution of balloons and, later, stents of increasingly sophisticated design and functionality. However, the mechanics of delivering these life-saving devices to the target lesion has largely remained unchanged—the interventionalist must manually thread a coronary guidewire through the peripheral vasculature to the diseased vessel. Navigating the coronary guidewire through the turns, twists, and branches of the coronary vessels typically requires an iterative, trial-and-error process of advancing, rotating, and retracting the wire until the target vessel is reached. The safety, speed, and efficiency of the procedure must be balanced delicately and can vary widely depending on the interventionalist's experience and procedure volume.

Robotic systems represent the newest tool in the armamentarium of the interventionalist [2, 3]. Serving as an extension of the skilled operator's hands, they translate manual movements of the guidewire, guide catheter, and rapid-exchange device into precise, millimeter-scale, robotically guided ones. The CorPath® GRX System (Corindus Inc., Waltham, Massachusetts, United States of America) is currently the only CE Mark-approved and FDA-cleared robotic platform for robotic-assisted percutaneous coronary and peripheral vascular procedures [4, 5]. In its ongoing effort to improve the features, functionality, and utility of the CorPath platform, the manufacturer has recently added a novel, optional automation to the platform's guidance software. Known as the “Rotate-on-Retract” feature, its function is to rotate the guidewire whenever it is retracted by the operator, changing the guidewire tip's orientation in preparation for the next advancement. By relieving the need for the operator to perform this action, Rotate-on-Retract facilitates faster and more precise maneuvering of the guidewire to the target lesion. The case presented here represents our experience with the first-in-human use of this new, automated guidance feature.

## 2. Case Presentation

In March of 2018, a 62-year-old male with a history significant for hypertension and dyslipidemia, and a body-mass index of 30.4, presented with symptoms of acute myocardial infarction. After counseling and informed consent, the patient was treated with a staged treatment approach, beginning with PCI and stent placement in the codominant right coronary artery (RCA). He was asked to return three weeks later for angiography and robotic-assisted PCI to the left anterior descending (LAD) coronary artery.

Angiography confirmed the presence of a patent stent in the RCA, a class IIB severe, long, diffuse, and calcified lesion of the proximal to mid LAD segments (90% stenosis; TIMI grade 1 flow). A severe lesion of the first obtuse marginal artery was also identified.

Prior to the procedure, the patient was sedated and an appropriate local anesthetic was applied at the radial access site. Clopidogrel, acetylsalicylic acid, and heparin were administered. Preparation for the robotic PCI procedure involved manual introduction of a radial access guide catheter (6F, EBU 3.5; Medtronic, Minneapolis, MN) and cannulation of the LAD according to standard practice. The guide catheter was then connected to the Y-connector, which was loaded into the appropriate drive of the robot's single-use cassette. A coronary guidewire (Balance Middleweight, 0.014 × 190 cm; Abbott Vascular, Abbott Park, Illinois, USA) was then introduced through the Y-connector into the guide catheter, and the distal end was also loaded into the cassette's guidewire track.

With set up of the robotic system complete, the operator was seated at the radiation-shielded workstation and began advancing the guidewire to the LAD under fluoroscopic guidance, employing the joystick controls on the robotic console, with the Rotate-on-Retract feature enabled. On initial advancement of the guidewire toward the LAD, the wire went into a diagonal branch. It was retracted and readvanced, and it went into another new branch. Following the second retraction, the guidewire moved forward to enter the target lesion (refer to the Supplemental Video (available here)). Total time for guidewire placement was a few seconds. Once the guidewire was in position at the target lesion, a rapid-exchange coronary angiography balloon was loaded and advanced for predilation using the robotic system.

**Figure 1:**
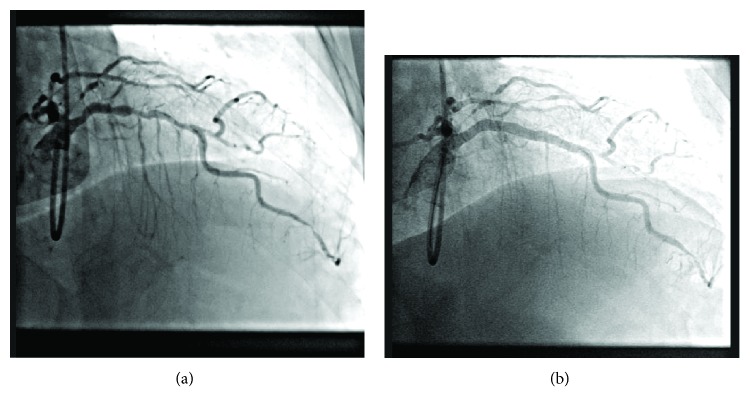
Intraprocedural angiography showing LAD before (a) and after (b) robotic-assisted PCI.

A rapid-exchange system for the delivery of two everolimus-eluting stents (XIENCE, Abbott Vascular) was subsequently loaded, delivered, and deployed using the robotic system. One 3.00 mm × 23 mm stent and one 3.50 mm × 28 mm stent were delivered to the mid and proximal LAD segments, respectively. Total procedure time was 1 h 55 min with 19.9 minutes of total fluoroscopy time. The entire PCI procedure was performed using the robotic system, and there were no intraprocedural dissections, perforations, device malfunctions, or other complications. At the conclusion of the case, vessel patency was confirmed by angiography with no evidence of residual stenosis and angiographic flow of TIMI grade 3 (Figure 1).

The patient returned later for scheduled PCI to the first obtuse marginal artery. He experienced a complete recovery.

## 3. Discussion

The evolution of minimally invasive, percutaneous interventional procedures has revolutionized cardiac medicine and surgery, vastly improving patient safety and outcomes after a wide variety of cardiac and peripheral procedures [1]. A great deal of energy and resources have successfully been invested in improvements focused on patient safety and outcomes. This success has provided the opportunity to now pay increasing attention to workflow and procedural efficiency, as well as the safety of clinical personnel.

We have been using the second-generation CorPath GRX platform at our institution for 7 months, for a variety of procedures including wiring side branches, main-vessel circumflex, and LAD with steep-angle issues. To our knowledge, this is the first report of first-in-human use of the new Rotate-on-Retract automation. Since this case, we have used it in at least 3 others. In our view, the principal advantage of the feature is that it automates a common maneuver, essentially combining the time usually required for two separate steps—retracting the guidewire and then rotating it—into one. Based on our experience with this feature, we believe that it has the potential to shorten wiring times, which is one of the more time-consuming parts of the PCI procedure, especially in the presence of tortuous vessels and challenging angulation. Indeed, preclinical data presented at the Transcatheter Cardiovascular Therapeutics (TCT) Conference in October 2017 demonstrated a 53 ± 23% reduction in wiring time (20 ± 8 sec versus 48 ± 21 sec; *p* = 0.03) when Rotate-on-Retract was enabled, even among operators who were highly experienced with robotic wiring [6].

We found it easy to adjust to the new feature. In this first case, manual rotation was employed a single time as we became familiar with its operation. Since then, we have found no need to use manual rotation in order to reach the target lesion. The automation has helped us navigate very quickly through what would previously have represented very challenging subtotal occlusions.

It is noteworthy that the use of the robotic-assisted PCI is also an advantage in terms of the operator's safety and well-being. While the robotic control of instrumentation affords greater control and millimeter-level precision to the operator, it also allows the procedure to be carried out remotely. Thus, the operator's ongoing exposure to intraprocedural fluoroscopy is reduced by shielding and physical distance, and at the same time, the need for heavy, orthopedically stressful protective equipment is eliminated.

In conclusion, we find that the CorPath Rotate-on-Retract feature is a safe and effective addition to the CorPath platform, improving the maneuverability of coronary guidewire movements. We believe it has the potential to shorten wiring times and enhance the overall efficiency of PCI procedures.

## Abbreviations

PCI: Percutaneous coronary intervention
LAD: Left anterior descending coronary artery
RCA: Right coronary artery.

## Supplementary Materials

Supplementary Materials Supplemental Video S1: navigation of the guidewire toward the LAD using the CorPath Rotate-on-Retract feature.
